# Modeling Inhomogeneous DNA Replication Kinetics

**DOI:** 10.1371/journal.pone.0032053

**Published:** 2012-03-07

**Authors:** Michel G. Gauthier, Paolo Norio, John Bechhoefer

**Affiliations:** 1 Department of Physics, Simon Fraser University, Burnaby, British Columbia, Canada; 2 Department of Oncology, Montefiore Medical Center, Moses Division, Bronx, New York, United States of America; 3 Department of Medicine, Albert Einstein College of Medicine, Bronx, New York, United States of America; Université d'Evry val d'Essonne, France

## Abstract

In eukaryotic organisms, DNA replication is initiated at a series of chromosomal locations called origins, where replication forks are assembled proceeding bidirectionally to replicate the genome. The distribution and firing rate of these origins, in conjunction with the velocity at which forks progress, dictate the program of the replication process. Previous attempts at modeling DNA replication in eukaryotes have focused on cases where the firing rate and the velocity of replication forks are homogeneous, or uniform, across the genome. However, it is now known that there are large variations in origin activity along the genome and variations in fork velocities can also take place. Here, we generalize previous approaches to modeling replication, to allow for arbitrary spatial variation of initiation rates and fork velocities. We derive rate equations for left- and right-moving forks and for replication probability over time that can be solved numerically to obtain the mean-field replication program. This method accurately reproduces the results of DNA replication simulation. We also successfully adapted our approach to the inverse problem of fitting measurements of DNA replication performed on single DNA molecules. Since such measurements are performed on specified portion of the genome, the examined DNA molecules may be replicated by forks that originate either within the studied molecule or outside of it. This problem was solved by using an effective flux of incoming replication forks at the model boundaries to represent the origin activity outside the studied region. Using this approach, we show that reliable inferences can be made about the replication of specific portions of the genome even if the amount of data that can be obtained from single-molecule experiments is generally limited.

## Introduction

**Figure 1:**
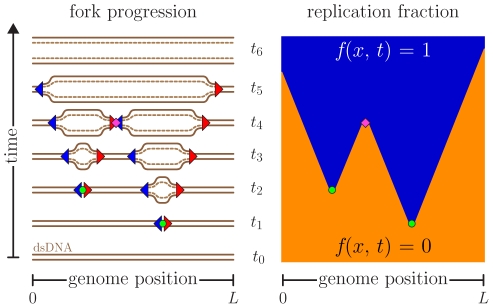
Space-time representation of the replication kinetics. The left-hand side shows the original (solid lines) and new synthesized (dashed lines) DNA while replications forks (triangles) are moving. In this example, the forks originate from two origins (circles) that are initiated at times 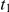 and 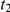. The forks move at a constant speed until they coalesce with another fork (diamond at 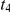) or reach the ends of the molecule of length 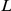 (around 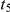 and 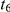). The right-hand side presents the space-time replication fraction 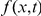, where 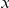 is the position along the genome, of the same replication cycle. Orange and blue areas represent unreplicated (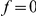) and replicated DNA (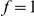), respectively.

Cells must accurately duplicate their DNA content at every cell cycle. Depending on the organism, the process of DNA replication can initiate at one or multiple sites called origins of replication. The DNA is copied by a pair of oppositely moving replication forks that emerge from each origin. These forks actively replicate the genome away from the origin until they encounter another replication fork. DNA replication can thus be modeled as a series of nucleations, growth (perhaps including fork stalls and rescues [1], [2]), and coalescences occurring in an asynchronous parallel way until the whole genome is copied [3], [4] (Fig. 1).

The complexity of the replication process traces back to the observation that the initiation program can be inhomogeneous in both space and time (see [5]–[11] for examples). Spatially inhomogeneous replication firing can be caused by a variety of factors such as an inhomogeneous distribution of pre-replication complexes or their uneven activation during the S phase. This is believed to be caused by factors such as the primary sequence of DNA, the presence of transcription factor binding sites, the chromatin organization of the DNA template and by gene expression [5], [12], [13]. The variability of origin initiation times, on the other hand, can result from the stochastic recruitment of replication initiation factors and the level of checkpoint activity [14]–[16]. As a consequence of such stochastic initiation, replication origins can also be passively replicated by forks coming from neighboring origins. In summary, modeling DNA replication is challenging because the probability of initiation of an origin varies along the genome, the moment at which an origin fires is stochastic, and origins do not systematically fire at each cell cycle.

DNA replication modeling is also challenged by the lack of direct observations. Experimental techniques using immunofluorescent labels to observe the DNA synthesis provide only snapshots of the replication kinetics [17]. The modeling approach presented in this paper can be used to reveal the detailed replication program responsible for producing these snapshots (initiation rates, fork speeds, stalling events, etc).

Over the last decade, our group has developed an analytic approach to modeling DNA replication kinetics [3], [4], [18]–[25]. The approach is based on a formalism inspired by the Kolmogorov-Johnson-Mehl-Avrami (KJMA) theory of phase-transition kinetics in one spatial dimension [26]–[30]. In general, this approach has assumed that there was no significant spatial variation along the genome in the parameters characterizing replication. (Except for Ref. [18] in which we looked at replication in budding yeast, where origins have fixed locations. Reference [18] turns out to be somewhat different from the present case, where origin initiation occurs in extended zones that then show variation along the genome.) In particular, we assumed that origin initiation rates and the rate of DNA synthesis (fork progression velocities) were spatially uniform. Temporal variations, however, were included, and their effects can be important [18], [20], [22]–[24]. Because our approach gives analytical solutions for the evolution of experimentally measurable quantities such replication progress, fork densities, domain densities, and the like, it is particularly well suited for fitting to experimental data [18], [20]. This offers an advantage compared to other approaches based on lengthy Monte Carlo simulations [31]–[35] because it requires far less computational power to fit experimental data.

In this paper, we generalize our analytic approach to the case where initiation rates and fork velocities may vary in both space and time. We derive simple rate equations that can be solved numerically to obtain the mean-field space-time replication kinetics. We find the average fork densities in both directions, everywhere along the genome and at any moment during the synthesis (S) phase of the cell cycle. This technique can be used to analyze experimental data from molecular combing [36], [37] and microarrays [38]–[40]. In addition, since our approach allows us to determine quantities involving DNA replication initiation, progression and termination (e.g., coalescence probability profiles, replication time distributions, etc.), it is particularly suitable for fitting results obtained from experiments based on the single-molecule analysis of replicated DNA (SMARD) where molecules at all stages of DNA replication are considered and the steady-state distribution of replication forks can be determined within a specific portion of the genome [7]. On the other hand, the mean-field assumption assumes that the cell-to-cell variations in parameters relevant to replication are small. It also does not give the statistical variation expected from an analysis of a finite number of cells, even when all cells are identical. Both of these limitations can be addressed by Monte Carlo simulations, which should be seen as complementary to the present approach.

## Methods

### Simulating DNA replication

Although the goal of this paper is to be able to calculate the average replication kinetics without recourse to numerical simulations, we shall use simulations here to test our model solutions and, more extensively, to test our fitting procedures. As illustrated in Fig. 1, we model DNA replication using a series of origins from which a pair of replication forks emerge to bidirectionally duplicate the DNA. These forks move away from the initiation site until they coalesce with another fork or reach the end of the molecule. At this level of description, only the rate at which forks are initiated, 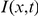, as well as their propagation speed, 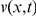, is needed in order to simulate the process of DNA replication. We previously used a Monte Carlo simulation to study the case in which origin initiation rates and fork progression are spatially homogenous along the genome, i.e., 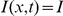 and 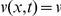. Such processes are described in detail in Ref. [3]. However, experimental observations indicate that initiation rates can vary in both space and time along the genome and that the speed of replication forks is not necessarily uniform. Hence, the Monte Carlo simulations must be modified to model these inhomogeneous factors. In addition, since in mammalian cells initiation events frequently appear scattered across large genomic regions (rather than being limited to the precise DNA sequences), we included in our simulations the presence of initiation zones. We chose Gaussian profiles for the zones. Although the form of such zones is not clear experimentally, our formalism can work with zones that have an arbitrary initiation-rate profile along the genome (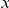-axis).

**Figure 2:**
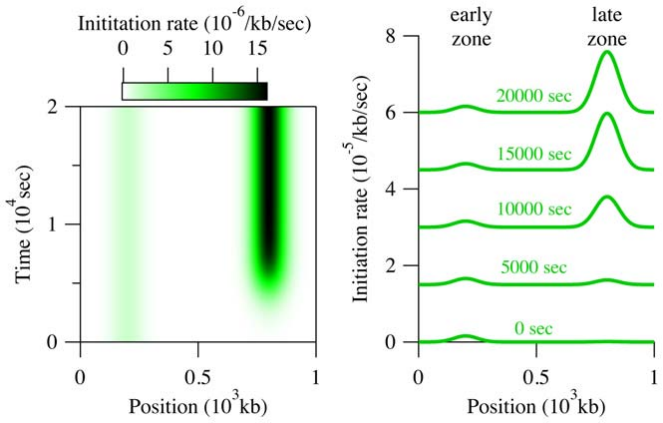
Initiation profile 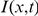
 **used to produce the results presented in** **Fig. 3.** The left-hand side is a density plot of the initiation rate, while the right-hand side shows 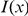 at various time points. (For clarity, each curve is offset by 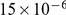/kb/sec from the previous one.) The initiation pattern is composed of two Gaussian initiation zones at 200 and 800 kb. The first, or “early,”zone is constant throughout time, while the second, more efficient, “late” zone is turned on at 5000 sec.

As a test case for our new model, we simulated the replication of a genomic region of 1000 kb containing two Gaussian initiation zones of similar size (50 kb), as indicated in Fig. 2. Each zone is assumed to contain origin that fire at different times during the S phase and therefore referred to as “early” and “late.” In Fig. 2, the early zone is centered at 200 kb and is active at all times. The late zone, located at 800 kb, on the other hand, is active only for 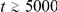 sec. The late zone is also assumed to be 10 times more efficient than the early one (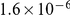 initiations/kb/sec at the peak of the early zone). The initiation rate 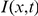 indicates the average number of initiations that occur at 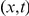 per length of unreplicated DNA per unit of time. This definition is motivated by the observation that each portion of the genome replicates only once per S phase. For this specific example, we set the fork velocity profile, 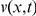 to a constant value of 0.04 kb/sec. The simulation parameters chosen here are typical of replication in somatic cells in mammalian organisms [41]. Our method can easily include variable velocities, including fork blocks due to DNA damage. (Our approach allows both *I* and *v* to be space-time dependent but the results of our test case are easier to interpret when there is only one inhomogeneous contribution to the replication kinetics.) However, experimental results indicate that the effects of the inhomogeneity of 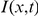 are much more important than the effects of the inhomogeneity of 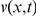 (see below and Demczuk *et al.*, unpublished). For simplicity, we used periodic boundary conditions (PBCs) for the fork propagation in our simulations (forks reaching a boundary are re-inserted at the other boundary). Therefore, it is formally equivalent to a circular chromosome (e.g., as in bacteria). Of course, whole-chromosome simulations of eukaryotic chromosomes would not use periodic boundary conditions and would take into account the specific (low) initiation rates found in telomeres [38].

**Figure 3:**
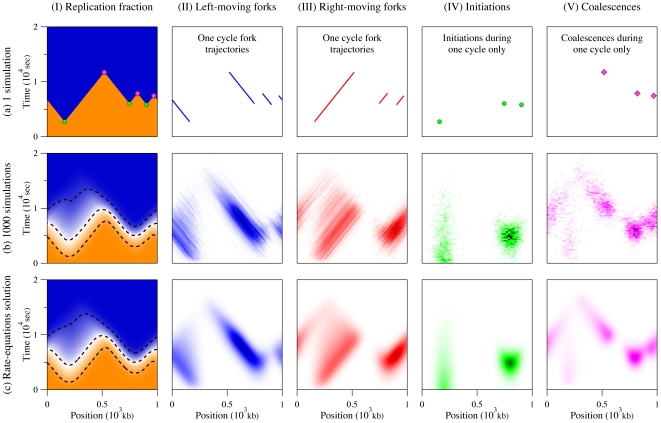
Comparison between one simulated replication cycle (a), 1000 simulation cycles (b), and our rate-equation solution (c). In graph (I), the color scale goes from 0 (orange) to 1 (blue); in graphs (II) and (III), it goes from 0 (white) to 0.01/kb (black); in graphs (IV) and (V), it goes from 0 (white) to 1.5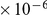/kb/sec (black). In all cases, we used the initiation function 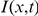 presented in Fig. 2 and the fork velocity 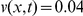 kb/sec. The genome size is 1000 kb, with periodic boundary conditions. Column I compares the replication fraction 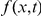 in the three cases. The dashed lines in b–I and c–I show the 10%, 50% and 90% contour curves. Columns II and III present the fork densities 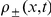. Fork densities are expressed in forks/kb in (b) and (c) while trajectories only are shown for the single cell cycle in (a). Columns IV and V present the space-time probability density functions of observing an initiation, 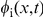, or a coalescence, 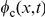, respectively. Part (a) shows where and when initiations and coalescences from one cycle occurred while parts (b) and (c) represent probability densities in 1/kb/sec.

Simulation results for our model system for 1 and 1000 cell cycles are presented in Figs. 3 a and 3 b, respectively. Each figure is divided into five parts. Part I shows the replication fraction 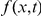. For the one-cycle simulation, the value of 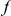 is either 0 or 1 for unreplicated and replicated DNA. For a finite number of simulations, as in Fig. 3 b–I, the value of 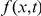 is the average value observed throughout the ensemble of simulated cycles (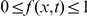). The fraction 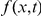 thus gives the probability that a specific section of the genome located at 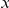 is replicated a time 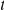. Parts II and III present the left- and right-moving replication forks. Only the trajectories of the forks are displayed for the case of one simulated cycle, while the average observed fork densities are reported for the case of many cycles. We refer to the fork densities presented Fig. 3 b–II and 3 b–III as 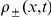, where the 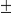 sign represents the right- and left-moving forks, respectively. These densities equal the number of forks moving in a specific direction per kb at 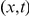. Finally, parts IV and V show where and when initiations and coalescences occur. In b–IV and b–V, these events are represented using probability density functions, 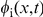 and 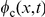, for initiations and coalescences, respectively. These densities are expressed in units of 1/kb/sec and are normalized so that 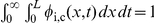.

Our simulations give detailed information about the replication process and its statistics. Typical quantities of interest that we study include the distribution of whole-genome replication times, the average replication times of different regions, and the average number of initiations and coalescences (as well as their space-time distributions). However, while simulations based on a known scenario can reproduce any experimentally obtainable statistic, the calculation times are long. To fit unknown parameters to a set of experimental data would require large computational resources. This difficulty motivates the analytic methods presented in the next section. Although they use numerical methods to solve differential equations, they are orders of magnitude faster than simulation-based approaches.

### Rate-equation approach

As mentioned above, we have developed a theoretical approach that can be substituted for numerical simulations in order to speed up the analysis of a given replication scenario when one is interested in the average replication kinetics. As we will show, integrating our rate-equations system also involves numerical steps, but our approach is still considerably faster than simulation-based models. Moreover, our method directly gives the mean-field kinetics of replicating DNA. This solution is equivalent to the simulation results in the limit where an infinite number of simulations is performed. (Compare Fig. 3 b to Fig. 3 c). In this sense, our technique provides the exact average replication program but does not give information about the cell-to-cell variability of the process, which can be obtained from simulations. Simulations are thus complementary to the present mean-field calculation method.

In this section, we introduce an analytical formalism to model the creation, propagation and annihilation of replication forks during DNA synthesis. To proceed, we derive a set of coupled differential equations that describe the change in the replication fork populations as a function of both the position along the genome (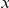) and the time since the beginning of S phase (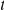). As before, we define 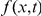 to be the probability that a given position of the genome 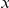 is replicated at time 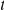, while 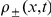 represents the right- and left-moving fork densities.

#### Modeling the replication kinetics using rate-equations

We describe the space-time evolution of the average replication fraction 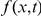 as well as both average fork densities 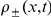, assuming that the creation and propagation dynamics of the forks are inhomogeneous, i.e., that the initiation function 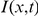 and the fork speeds 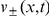 can vary in space and time. (Again, the 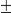 signs refer to the direction of propagation of the forks.)

The first equation of our set gives the rate of change of the probability that a given location, 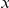, is replicated at time 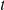,
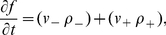
(1)which is simply given by the product of local fork densities times the rate at which a given fork synthesizes DNA.

The rate of change of the fork densities can be expressed in the form of a “transport” equation,
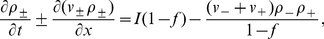
(2)with a “source” and a “sink” term on the right-hand side. The source term, 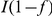, represents the initiation of new forks at a rate 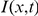 rescaled by the probability that the genome is not already replicated at that position, 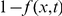. The sink term represents the annihilation rate of forks as they coalesce with oppositely moving forks. The coalescence rate is proportional to the two local populations of forks and the relative speed at which these forks are merging. That rate must be normalized by the probability of not being replicated, 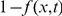. The 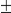 sign on the left-hand side of Eq. 2 arises because both left- and right-moving forks are assigned positive velocities. An expression similar to Eq. 2 has been used to model the growth of crystal lamella [42].

Given a replication scenario for 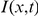 and 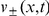, Eqs. 1 and 2 can be numerically integrated to obtain 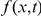 and 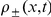. We solved our set of equations for the same conditions as used for the simulations presented in Fig. 3 b (i.e., 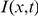 given by Fig. 2 and 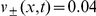 kb/sec). We explicitly integrated our equations using 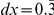 kb and 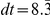 sec (we need 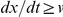 to adequately solve this system). We also used PBCs to solve our equations, which means we used 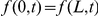 and 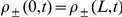 for all 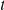. Using 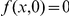 and 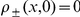 for all 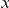 as initial conditions, the solution presented in Fig. 3 c agrees with the simulation results of Fig. 3 b within statistical limits. Parts I to III are directly obtained from the solution of our three rate-equations. The densities of parts IV and V are, on the other hand, proportional to the source and sink terms of Eq. 2 , respectively. Hence,
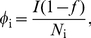
(3)where
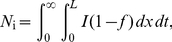
(4)is the average number of initiations per replication cycle. Similarly,
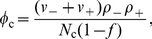
(5)where the average number of coalescences per cycle is given by
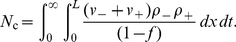
(6)The results of our numerical integrations are 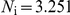 and 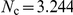. For a finite-size system with periodic boundary conditions such as the model presented in this paper, we must have 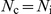. The 0.2% difference between our calculation results is simply due to round-off errors. Our calculation also matches the average number of 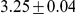 initiations observed during our 1000 simulations. Finally, note that our model can also be solved using non-periodic boundary conditions in order to study replication of linear DNA. In such a case, the numbers of initiations 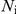 and coalescences 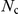 are still given by Eqs. 4 and 6, but we expect 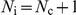.

#### Start-time distributions

The stochasticity of the replication process modeled here implies that the start and end of S phase (defined by the first origin initiation and the last fork coalescence) occur at different times each single cycle. As illustrated in Fig. 3 a–I, the simulation starts at 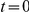, but the actual duplication of the DNA does not start before 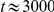 sec. In other words, there is a distribution of replication start times (marked by the first initiation) and also a distribution of end times (marked by the last coalescence).

**Figure 4:**
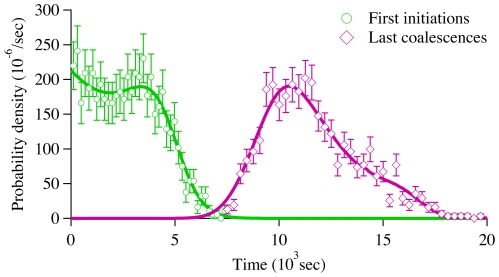
Replication starting and ending times density functions, 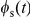
 **and** 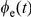 **, for our model system.** Symbols were obtained from simulations, while solid lines were calculated from the solution of our rate-equations.

Our rate equations can be used to calculated the probability that replication has started, 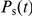, as a function of time, which corresponds to the probability that at least one initiation occurs during the time interval 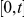. We calculate this probability in terms of a related quantity, 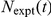, which is the number of initiations that are expected to happen in 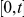, assuming that there were no initiations prior to 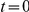. We write
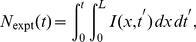
(7)where 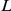 is the genome length. Consequently, the probability that at least one initiation occurred prior to time 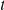 is given by
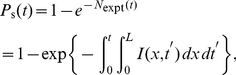
(8)and the replication starting time distribution is simply given by 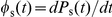. Figure 4 compares the calculated starting time distribution with simulation results.

Equation 8 is valid for any molecule of length 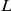, whether periodic or non-periodic boundary conditions are considered. However, Eq. 8 must be modified if one is studying a finite-size fragment that is part of a larger molecule. A fragment thus corresponds to a finite-length linear molecule without PBCs but with a flux of forks at its boundaries (these forks were previously initiated elsewhere outside the fragment region). In order to calculate the starting probability of such a fragment, we first define the notion of *directional replication fractions*, 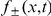, which are the probabilities that the location 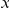 has been replicated by a right- or a left-moving fork. These replication fractions are obtained from
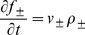
(9)and can be calculated as a by-product of the numerical integration of Eq. 1 . Thus, for a fragment that begins at 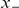 and ends at 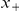, the probability that replication has started is given by
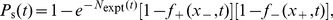
(10)where
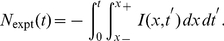
(11)Equation 10 says that the probability that replication has not started along a given molecule is the product of the probability that no initiation already occurred within the molecule times the probability that no fork came across the molecule boundaries.

#### End-time distributions

Another useful quantity is the probability that replication has ended at time 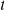, 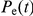. This quantity is of great interest because it tells us when the replication is over. It could therefore be used to study the duplication time of the genome as a function of the replication scenarios. In general, we cannot derive an analytical solution of our nonlinear rate-equations system and, consequently, we cannot derive a formula for 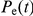 as we did for the starting-time distribution. Nonetheless, we can use our knowledge of the replication fork density to estimate 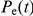 as the probability that there is no fork along the genome. For a periodic system, where the number of right-moving forks is always equal to the left-moving forks, we have
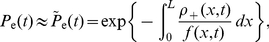
(12)where we have assumed the number of forks at time 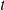 to be given by a Poisson distribution (an equivalent estimate for *P_e_*(*t*) in a system with PBCs is obtained by replacing *ρ*_+_ (*x*; *t*) by *ρ*_−_ (*x*, *t*) in Eq. 12). The tilde notation used in Eq. 12 denotes the fact that 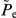 is an approximation of 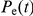. The density is normalized by the probability of being replicated. Figure 4 also compares the end-time distribution function, 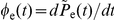, with simulation results. Note that we can replace 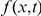 by 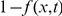 in Eq. 12 to get an estimate of 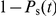. We expect the approximation that fork distributions are Poisson to be accurate at the beginning and end of S phase, where the number of forks is small, but to be less so in mid-S phase, where there are more forks. For the model explored here, the maximum difference between the calculated and the simulated values of the ending probability is 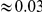. In Supporting Information S1, we solve exactly the case of a uniform initiation profile and show that the error of our approximation of 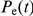, when compared to the exact solution, decreases as the number of initiations increases (i.e., as 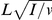 increases).

In the case of a non-periodic system, the lack of forks that move in one direction over a certain range 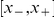 does not imply that the whole range is replicated. Therefore, we must modify Eq. 12 so as to obtain the end-time distribution of finite-size systems without periodic boundary conditions (e.g., finite-length linear DNA or a section of a larger molecule). The probability that a DNA fragment located between 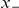 and 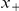 is fully replicated is given by
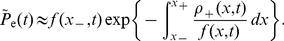
(13)Equation 13 asserts that the replication of a molecule without PBCs has finished if no right-moving forks are observed and if the left boundary is replicated (or vice versa for left-moving forks). As mentioned above, an equivalent estimate for *P_e_*(*t*) without PBCs is obtained if we substitute the pre-factor f(*x*_−_, *t*) by f(*x*_+_, *t*) and use *ρ*_−_ (*x*, *t*) instead of *ρ*_+_(*x*, *t*).

#### Boundary fork injection

The previous sections presented how our model can be used to study replication of molecules with and without PBCs. Deriving Eqs. 10 and 13, we even demonstrated how to calculate the probability that a sub-section of the modeled systems has started or ended replicating. Here we now show how we can adapt the boundary condition so they act as sources of forks in order to account for initiations that occur outside the modeled DNA segment. These forks mimic initiations occurring outside 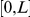. The simplest case would be to have a source term that is equivalent to a semi-infinite region where the initiation rate and the fork velocity are constant. In such a case, the density of forks at the boundaries is simply
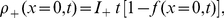
(14)

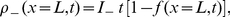
(15)where 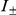 are the constant initiation rates outside the modeled regions (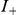 for right-moving forks coming from the 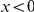 region and 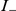 for left-moving forks initiated at 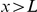). The derivation for this boundary condition is presented in Supporting Information S1 and the Figure S1.

#### Stochastic fork progression

Our calculation method can also be adapted to model the impact of DNA damage on replication kinetics. Even in normal, healthy cells, there are a large number of DNA “defects” where forks slow, or even stop. Such damage usually affects only one of the two DNA strands. These single-strand lesions are characterized by base oxidation caused by reactive oxygen species or by base misincorporation due to a copying error during DNA replication. In more serious but rarer cases, defects involve both DNA strands. Examples of such double-strand defects include DNA crosslinking induced by ionizing radiation or double-strand breaks that result from a failed repair to single-strand damage. Double-strand damage is more dangerous because its repair can lead to rearrangements of the genome and even contribute to the development of cancer [43]. Depending on their density and on the repair mechanisms involved, DNA damage can have a strong impact on the replication kinetics. The slow down or stalling of forks at defects gives more time to fire to origins that would otherwise have been passively replicated [44]. Also, fork stalls trigger local and global checkpoint signals that can affect the progression of forks and the firing rate of new origins elsewhere along the genome [11].

If replication speed changes predictably along the genome, one can simply define an appropriate velocity profile 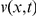. However, fork progression can also be affected in a more stochastic way in the presence of DNA damage. When they encounter such defects, replication forks are stalled for a given period of time until repaired. The repair time depends on the nature of the defects and can either be finite or infinite (i.e., not repaired during the current S phase). In the infinite-repair-time case, the replication of the DNA on the other side of the defect must come from the oppositely moving fork. Such a stochastic blocking/unblocking mechanism can be added to our mathematical framework by modifying our expression for 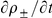 to
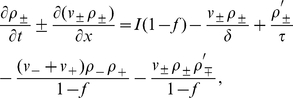
(16)where the five terms on the right hand side are

1. the initiation rate of new forks, as we had in Eq. 2 ;
2. the stall rate of the moving forks, assuming that the average spacing between defects is given by 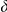;
3. the repair rate of the stalled forks, denoted 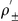, with the average repair time given by 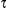;
4. the coalescence rate between moving forks, per Eq. 2 ;
5. the coalescence rate of moving forks that collide with stalled forks.

The densities of stalled forks can be obtained by adding two differential equations to our set. These new equations are used to describe the rate of change of the densities of forks that are stalled at DNA lesions as
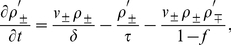
(17)where the three terms represent stall, repair, and coalescence rates. There is no 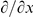 term on the left-hand side of Eq. 17 because stalled forks are assumed to be fixed in space. A simplified version of this fork-stall model, neglecting spatial inhomogeneity, was the subject of a previous publication [19].

## Results

### Analyzing experimental data

An obvious application of our analysis would be to reproduce results from experiments based on microarrays [38]. Microarrays provide genome-wide average replication profile as a function of time (derived from the overall molecule replication fraction), which ideally corresponds to the replication fraction 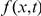 obtained from our rate equations (see [39], [40] for examples). Of course, real microarray experiments are not ideal, and issues such as the spatial resolution of the array or the cell-cycle asynchrony of populations should be kept in mind when analyzing the data. In a future contribution, we shall discuss how to reproduce such time-course results. Here, we demonstrate the versatility of our modeling technique by adapting it to the study of a more subtle type of data that has recently been obtained via single molecule analysis of replicated DNA (SMARD), a method developed by Norio *et al.* [7]. The modeling and fitting procedures presented in this paper were used to analyze a large SMARD data set obtained from mice bone marrow cells (Demczuk *et al.*, unpublished). One feature of such experiments is that the data are obtained from an asynchronous population of cells (i.e., the starting time of each cell in the population is random, drawn from a uniform distribution). Unlike microarrays, SMARD also allows one to determine the steady state distribution of replication forks, as well as the location of initiation events and fork collisions (in addition to the temporal order of replication for a specific portion of the genome). This additional information can be used to determine more precisely the level of origin activity across the genomic region analyzed. We shall need to adapt our model to make predictions for such a case.

#### Simulating a SMARD data set

The goal of the current section is to adapt our calculation approach to the analysis of an actual experimental setup, the SMARD experiment. The first step towards such a goal is to be able to simulate the data collected during this experiment.

**Figure 5:**
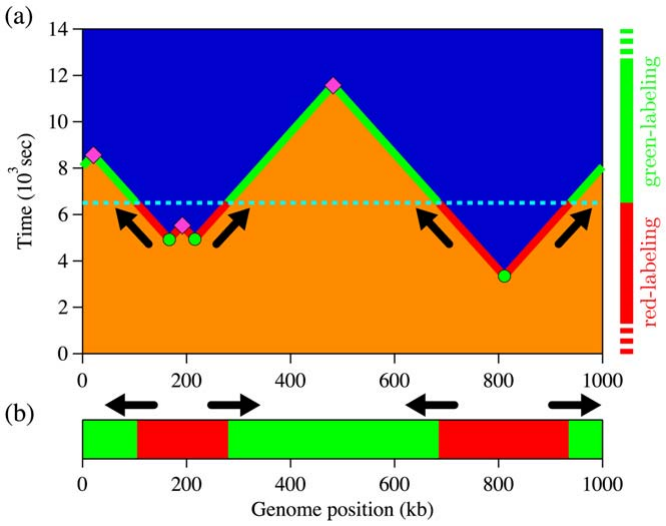
SMARD labeling procedure. (a) Example of a replication space-time profile and the corresponding SMARD labeling procedure. As before, blue sections indicate replicated DNA while orange sections represent unreplicated DNA. Circles denote fired origins, while diamonds indicate coalescences of replication forks. Periodic boundary conditions were used (circular genome). The dashed line at time 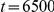 sec indicates the end of the first labeling period (red) and the beginning of the second (green) one. Arrows indicates the fork propagation directions at the labeling transition time. The labeling timeline on the right side and the solid line on the space-time profile illustrate the labeling process to produce the molecule example presented in (b). (b) Example of a molecule extracted from the simulation presented in (a). Red sections were replicated during the red pulse (before 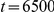 sec), while green sections were replicated later. To obtain a two-color molecule, the label transition time must occur after the first initiation and before the last coalescence.

The SMARD procedure is presented in detail in Ref. [7]. Here, we give a brief summary. In a population of asynchronously growing cells, one supplements the normal nucleotides used to synthesize DNA by two different types of halogenated nucleotides that are then conjugated to fluorescent antibodies. For convenience, we shall refer to them as red and green labels. (The first label is red; the second is green). Since cells are replicate asynchronously, the labeling switch can occur at any time relative to the cell cycle for a particular cell. (In particular, the switch will often occur when the cell is not in S phase.) Figure 5 depicts the labeling procedure when the transition happens during the replication process. Part (a) compares the labeling timeline with the replication space-time diagram, while part (b) shows the DNA molecule one would observed after such labeling. As shown in Fig. 5 b, the positions where labels are changing indicate the locations of the replication forks at the switching time (depicted by arrows). Then, if we know the labeling sequence (red followed by green in this case), we can distinguish left- from right-moving forks (forks are moving from red to green zones).

In practice, the red- and green-labeling periods are preceded by normal periods of non-fluorescent nucleotide synthesis. If each of these labeling periods is significantly longer than the duplication time of the analyzed molecules, then every molecule that is examined will show one or two types of nucleotide (but never three). All replicated molecules are collected, but only the ones that are fully labeled with fluorescent markers are kept for analysis (fully red, fully green, or red-green molecules).

**Figure 6:**
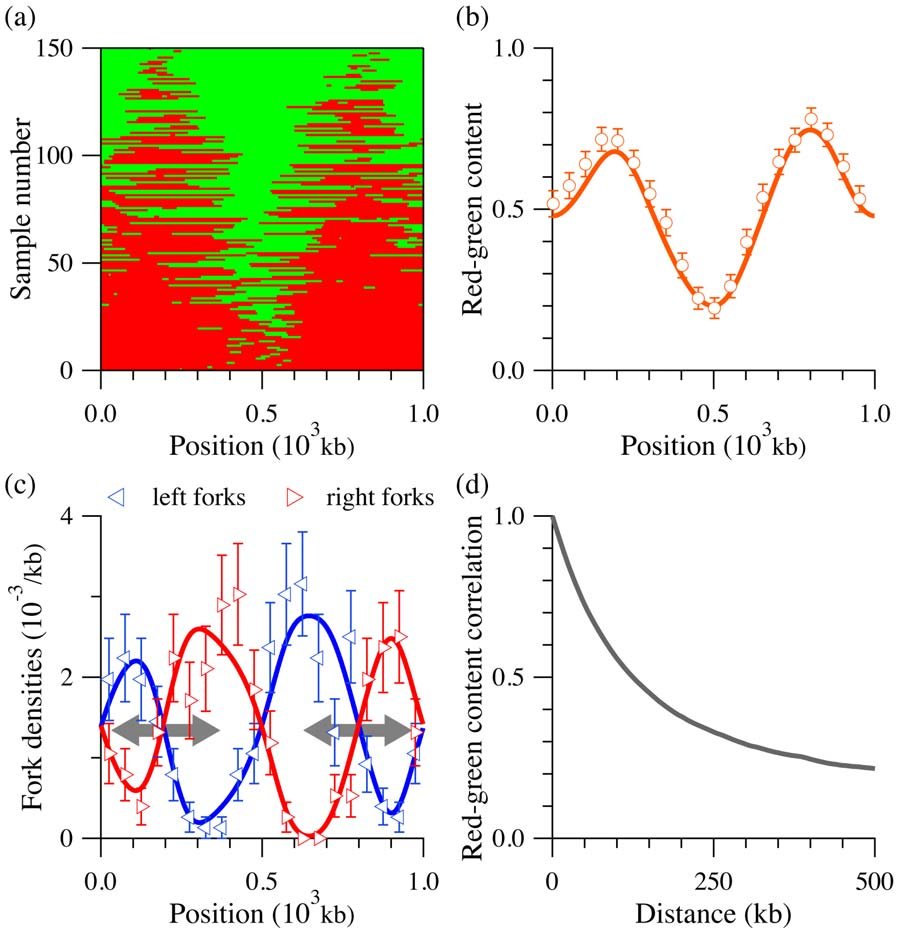
Simulation of SMARD experiment with comparison to rate-equation estimates. (a) Labeled molecules collected from simulations of the SMARD procedure, using the model system of Fig. 2 . Each line corresponds to a molecule as the example presented in Fig. 5 b. Molecules were organized according to their red-label content. Only molecules that were fully substituted with fluorescent nucleotides were considered for the analysis. (b) Red-green content 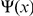 of the molecules from (a) as a function of the position 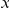 along the genome (circles). A value of one (zero) means that all the molecules are red (green) labeled at a given position. The solid line was calculated using our rate equations for 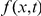 (see Eq. 23). Red-green content was determined by averaging over 5 kb bins; for clarity, only one value in ten is shown. (c) Left- and right-moving fork densities 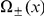 observed in the molecules presented in (a) as a function of the position 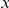 along the genome (triangles). The fork density is defined as the number of forks per unit length at a given position (using 50 kb bins, 10 times larger than the simulation bin size). The solid line is derived from the rate equations for 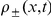 (see Eq. 24). Gray arrows in background show the locations of initiation zones (i.e., from left to right, the intersections of increasing right-moving fork densities with decreasing left-moving fork densities). (d) Autocorrelation function of average red-green content, computed from the pool of molecules presented in (a). Since we used periodic boundary conditions, the maximum displacement is 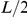.

The molecule-selection procedure described above–replication simulation followed by random molecule selection–can be repeated to collect a distribution of molecules. Figure 6 a shows an example of 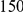 red-green labeled molecules collected during a simulation of our model system (Fig. 2) using the protocol of the SMARD experiment. We simulated more molecules but kept only the ones with both labels. The red-green molecules in Fig. 6 a are organized according to their red-label content. Note that a simple visual inspection of Fig. 6 a is sufficient to obtain a general sense about the position and relative efficiency of the replication origins located in the region.

#### Data analysis

Figures 6 b and 6 c present three statistical “profiles” that are functions of the genome position but averaged over all the simulated molecules shown in Fig. 6 a: the local red-green ratio and the densities of replication forks in both directions. Quantities are averaged over all samples because typical experimental data sets are small (10 to 100 red-green molecules, Demczuk *et al.*, unpublished). As we shall see in the next sections, we can adapt our approach to reproduce such average quantities without having to do simulations.

Figure 6 b shows the red-green content, 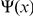, as a function of the genome position averaged over all the molecules collected in Fig. 6 a. This quantity is always between one (all red) and zero (all green) and is given by
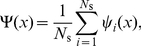
(18)where 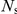 is the number of samples collected and 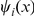 is the label value (1 for red and 0 for green) of sample 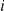 at the position 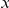. Figure 6 b clearly shows that the positions, widths and amplitudes of the red-green content function peaks correlates with the initiation zones in Fig. 2 . To a first approximation, a maximum of 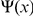 corresponds to an initiation zone, while its numerical value reflects the zone efficiency. We verified that an increase of the initiation zone width also correlates with an increase of the corresponding red-green peak width (not shown).

Another measurement that can be extracted from SMARD experiments is the position of forks along the genome. Figure 6 c shows the fork densities 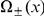 as a function of the genome position (again, the 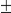 sign refers to right- and left-moving forks, respectively). Since the fork density is defined as number of forks per kb, it is, in the context of the SMARD experiment, given by
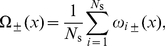
(19)where the local fork density 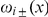 is the number of forks observed in sample 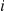 in a bin of size 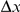, divided by 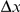. Again, the fork densities shown in Fig. 6 c were obtained from all the molecules presented in Fig. 6 a. These figures also show that the two fork densities can be used to characterize the initiation zones. For example, the position of an initiation zone approximately corresponds to the intersection of a decreasing left-moving fork density with an increasing right-moving fork density. Of course, since there are fewer forks per molecule, the fluctuations in densities are higher than the fluctuations in red-green content. Intuitively, this observation results from the fact that initiation zones are regions from which both types of forks emerge, leading to the observed positive and negative gradients of right- and left-moving fork densities across the zones. In other words, a right-moving fork is more likely to survive (not coalesce) as its moves across the zone (and vice versa for left forks). The converse situation, decreasing right-moving fork density and increasing left-density, characterizes termination zones, which are regions where coalescences are more likely to happen.

#### Estimating SMARD-like data from rate-equations results

Solving the rate equations (Eqs. 1 and 2) does not directly lead to quantities that we can compare to data obtained from SMARD experiments. The quantities 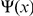 and 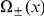 are not simple time averages of 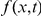 and 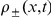. In the SMARD experiment, one collects only molecules with red and green labels, which means that all of them come from DNA that was replicated during the two labeling periods. For example, that means that fragments can only be collected between 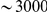 sec and 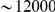 sec in the case illustrated in Fig. 5. However, the 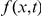 profile obtained from our rate equations corresponds to the average of an infinite number of space-time replication events similar to the one shown in Fig. 5 but it includes information collected at all times from 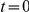 to 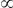. Consequently, the information prior to the first initiations and after the last coalescences that is incorporated in our rate-equation solution must be taken out to model the SMARD results. Fortunately, we can use our knowledge of the probabilities 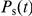 and 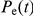 to estimate 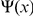 and 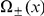.

In order to convert our calculated mean-field profile 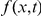 to SMARD-like red-green content function 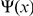, we first recall that 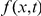 is the average of an infinite number of single replication events similar to the one depicted in Fig. 3 a–I (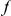 is 0 or 1 in Fig. 3 a–I, while it is a continuous number between 0 and 1 in Fig. 3 b–I and in 3 c–I). The replication fraction profile in Fig. 3 b–I is given by
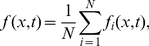
(20)where 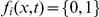 is a single-event replication profile (as in Fig. 3 a–I), and 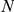 is the number of events (or simulations). The solution to the rate equations corresponds to 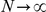. Equation 20 can be re-expressed as
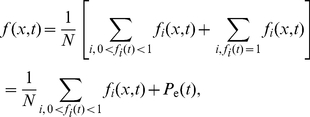
(21)where 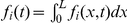 is the replication fraction averaged over the whole molecule. The terms with 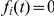 represent molecules collected at time 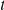 that have not begun to replicate. They are not included in the sum in Eq. 21 , since they each contribute 0. The terms with 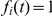 represent molecules collected at time 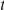 that have completely replicated. Their average just gives the probability that replication has ended by that time, 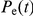.

Assuming the population of cells to be perfectly asynchronous, we can collect molecules at any time 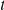, as long as replication has started, but not ended, at time 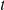. Consequently, our estimate of the red-green content function 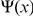 from the rate-equation solution is given by
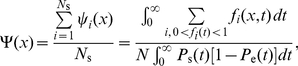
(22)where the number of samples 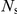 is given by the number of replication events 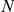 times the integral of the probability that DNA is actually being replicated at time 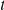 (i.e., the probability that replication has started multiplied by the probability that it has not finished). Using Eq. 21 , we can rewrite the red-green content function in a form that can be evaluated in terms of the rate-equation solution:
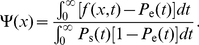
(23)Note that the term 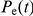 corrects for fully replicated molecules that are included in the calculation of 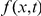 but not in 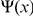. (No correction is needed for completely unreplicated molecules since their 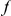-value is zero.) We use Eq. 23 and the solution to the rate equations to plot the solid line in Fig. 6 b.

Similarly, the average fork density in the SMARD experiment 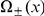 is given by
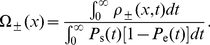
(24)After substituting the rate-equation solution into Eq. 24 , we plot the solid lines in Fig. 6 c. In contrast with Eq. 23 , no correction for fully replicated molecules is needed in Eq. 24 since fully replicated molecules have no forks (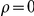).

Figure 6 b and c compare our calculated estimates of 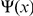 and 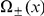 to simulation results. These figures demonstrate that Eqs. 23 and 24 can be used to accurately reproduce the simulated profiles obtained from experimentally typical size data set. Consequently, our model can be used to fit SMARD data in order to infer the initiation and fork velocity profiles.

One last issue that needs to be addressed is that the data points obtained from a single SMARD experiment are correlated. We can see this in Fig. 6 d, which plots 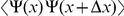, the autocorrelation function, as a function of 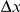. This means that the probability of being replicated at 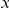 is not independent of the probability of being replicated at 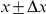. As a consequence, the weights given each point in a fit must take into account that errors in nearby points are likely to be similar in neighboring bins.

#### Fitting to correlated data

Standard least-squares fitting programs assume that the statistical errors in each data point in the fit are independent. However, we have just argued that our errors show significant correlations. In order to make valid inferences about issues such as the goodness of fit, we need to take these correlations into account. To do this using standard curve-fitting routines, we linearly transform the data set to diagonalize the covariance matrix (see [45] for example). Such decorrelated data are then independent, which means that standard statistical tests (e.g., the chi-square statistic) can be used to measure the quality of a fit. Moreover, as we shall see, the diagonalization can be done in a way that evenly weights all decorrelated data (i.e., the weights can be set equal to one). Equal weights are optimal numerically for curve fitting.

Let the experimental data be expressed as a one-dimensional vector 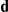 that comprises the red-green profile and the fork density densities (or any other information we can extract from both the data and our rate-equation solution). The covariance matrix 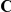 of the data set 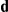 is then given by
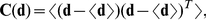
(25)where 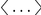 represents an ensemble average over many repetitions of the experiment. The decorrelation procedure requires a matrix 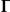 that changes coordinates in the data space so that 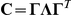, where the matrix 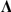 is diagonal. We say that 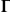 is a *decorrelation matrix* because the covariance matrix of the decorrelated data, denoted 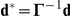, is given by the diagonal matrix 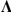. Given a correlation matrix 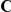, many different valid decorrelation matrices can be found, as long as 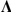 is diagonal.

We can restrict the choices of decorrelation matrices by adding the constraint that all the decorrelated data points should have equal weight. This means that the diagonal matrix 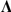 can be scaled equal to the identity matrix, which implies that the decorrelation matrix 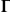 satisfies 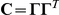. One way to obtain such a factorization of the correlation matrix is to perform a Cholesky decomposition of 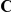 such that [46]

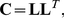
(26)where 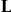 is a lower triangular matrix. The Cholesky decomposition can be performed on the correlation matrix because 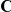 is, by definition, symmetric and positive definite. Consequently, the Cholesky matrix 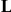 converts correlated data into evenly weighted decorrelated data (with all weights set to unity). Then, the following recursive procedure can be used to find the best fit of the data set:

1. Choose an initial replication scenario (initiation rate and velocity profile) that approximately reproduces the observed data 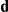. In order to perform a fit, the scenario must be expressed using a finite number of parameters.
2. Solve the rate equations using the current replication scenario. Estimate the data set 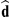, consisting of the red-green and fork-density profiles.
3. Perform 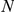 simulations based on the current replication scenario. Each simulation should collect the same number of fully labeled molecules as were collected during the real experiment. Analyze each simulation in the way real molecules were treated, and record the series of simulated data vectors 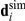, where the index 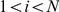.
4. Calculate the covariance matrix of the simulated data, 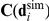. In practice, if the number of simulation runs is not large enough, the estimated covariance matrix may not be positive definite, as required to perform a Cholesky decomposition. Alternately, one can parametrize (e.g., by exponential decays) the correlations and fit any unknown parameters to simulation data. The form of the parametrized covariance matrix, denoted 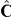, can chosen to ensure that 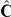 is positive definite.
5. Calculate the Cholesky decomposition matrix, 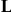, of the parametrized covariance matrix such that 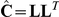 [46].
6. Decorrelate the observed data 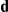 using the Cholesky matrix. The decorrelated data, denoted 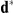, are given by 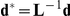.
7. Fit the decorrelated data 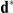 with the decorrelated solution of our rate-equations, 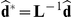. The fit searches for the replication scenario that minimizes the difference between the decorrelated data vectors 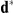 and 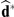 (where the weights of all data sets components are equal and set to unity). The correlated fit solution is given by 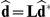.
8. Repeat, starting from Step 2, using the latest fit result as the current replication scenario, until the solution converges.

#### Fit example

We now apply the correlated data fitting procedure described above to a real SMARD data set. The data we use here and all the experimental details related to their collection can be found in Demczuk *et al.*, unpublished. In this paper, the SMARD technique was used to study DNA replication in mouse bone marrow pro-B cells at different developmental stages. The study was performed on four adjacent restriction fragments that cover about 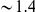 Mb of the genome. Because the fragments come from a much longer genome, we did not use periodic boundary conditions but instead modeled explicitly the injection of outside forks into the studied region.

**Figure 7:**
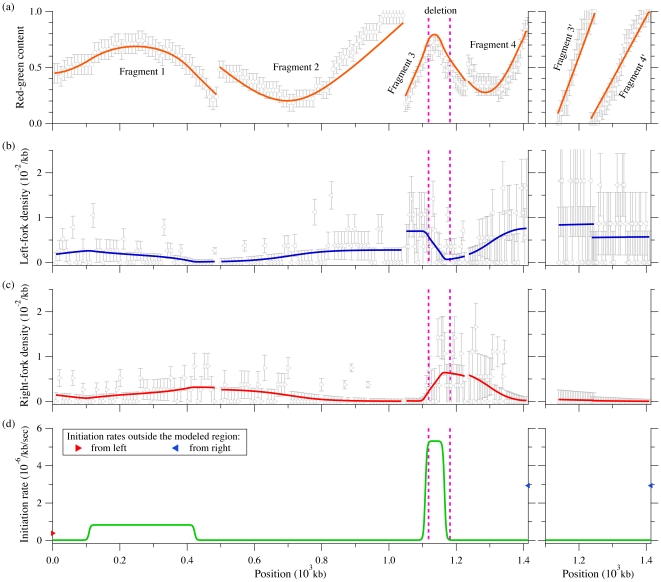
SMARD analysis of DNA replication in mouse bone marrow pro-B cells. The left side presents the data collected from four fragments covering a 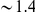 Mb region in normal cells. The right side shows data obtained from clone cells where the genome sequence was rearranged (65 kb was deleted from the genome). ÊThe deletion is located between the two dashed lines on the left side graphs. Only the equivalent of fragments 3 and 4 from normal cells was studied in the clonal population. Symbols represent experimental data while solid lines refer to the solution of our rate-equation system. (a) Red-green content 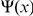 obtained from Eqs. 18 (symbols) and 23 (solid lines). (b, c) Left- and right-moving fork densities 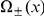 given by Eqs. 19 (symbols) and 24 (solid lines). (d) Best fit result for the initiation rate 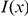 (solid lines) and boundary fork injection rates (symbols) used to solve our rate-equations. The best-fit fork velocities we obtained were 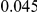 kb/sec and 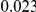 kb/sec for normal and clonal cell populations, respectively. Errors bars in (a, b, c) were obtained from simulations of the best-fit replication scenario.

In Fig. 7 , we present global fits to six different fragments (from Demczuk *et al.*, unpublished). The term “global” here means that all the fragments are simultaneously fit by a common, or global, set of parameters. Fragments 1 to 4 cover the studied region in unrearranged normal pro-B cells (left side of Fig. 7). The last two fragments (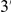 and 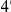) come from a clonal population of cells containing a genomic rearrangements within fragment 3 (right side of Fig. 7). The rearrangement of fragment 3 into 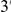 consist in a genomic deletion of approximatively 65 kb (located at 68 kb from the right end of fragment 3, see dashed lines in Fig. 7).

In fitting the experimental data, we made the following assumptions about the replication scenario:

1. Based on the normal cell red-green content profile (left side of Fig. 7 a), we assumed that two initiations zones are present (around 250 kb and 1150 kb). Each zone has three parameters that describe the position, width, and initiation rate of the zone. Another parameter defines a constant background of initiation (this parameter was added because low levels of initiations were observed outside the initiation regions). Finally, two other parameters describe fork injection rates at the boundaries of the modeled region (see filled symbols in Fig. 7 d).
2. For practical reasons, we assumed that the shape for the initiation zones was a rounded box, such as the ones shown in Fig. 7 d. As we see in Fig. 7 , the red-green content profile is not too sensitive to the precise shape of the initiation zones (e.g., the red-green content maxima have smoother edges than their corresponding boxy initiation zones).
3. We also assume that the initiation profile does not change with time during the S phase. Time-dependent profiles were considered but did not affect significantly the fit (unpublished observation).
4. Data sets from unrearranged and rearranged alleles were assumed to have the same initiation rates except within fragments 3/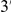. The linear red-green content profiles and the corresponding fork densities of fragments 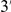 and 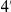 indicate that these fragments are almost always replicated by left-moving forks coming from the right side of fragment 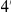. We thus assumed that the initiation profile of the deleted allele is the same as the one of undeleted allele except for the absence of the second initiation zone located within the deleted region (compare fragments 3 and 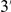 in Fig. 7 d).
5. We assumed a constant velocity throughout the four fragments. However, the experimental results presented in Demczuk *et al.*, unpublished, indicate that forks propagated at different speeds in these two experiments (probably caused by differences in the growing rate of the cultured cells in the two experiments). Therefore, we used two fork speed parameters, one for fragments 1 to 4 and another one for fragments 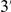 and 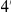.

The hypothetical replication described above comprises 11 free parameters that can be adjusted throughout a fitting routine (6 for the two initiation zones, 1 for the background initiation rate, 2 for forks coming from outside the modeled region, and 2 for velocities in both cell types). Using that hypothesis, we followed the fitting procedure described in Section to perform a global fit of the SMARD data collected from the six fragments. The fitted 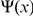 and 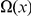 profiles are shown as solid lines in Fig. 7 a–c. The best-fit results are illustrated in Fig. 7 d as an initiation-rate curve. Note that our rate-equation system has to be solved two times for a given set of parameters (with and without the second initiation zone for normal and clone cells, respectively).

Since determining the replication program was the aim of the experiment, the quality of the fit cannot be directly compared to the “actual” replication program. However, SMARD provides information that was not used for the fit. Hence, it is possible to verify that the result of the fit are consistent with this additional information. First, the fitted fork velocities we obtained are 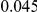 kb/sec and 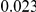 kb/sec (both 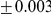 kb/sec) for the normal and clonal data set. The corresponding experimental values are 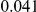 kb/sec and 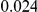 kb/sec (Demczuk *et al.*, unpublished). Considering the small sample sizes used to obtain these fork velocities (from 11 to 57 fully labeled molecules only, depending on the fragment, Demczuk *et al.*, unpublished), we evaluated from simulations the statistical errors for the measured fork velocities (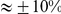). (Experimentally, the fork velocity within a fragment is calculated as 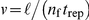, where 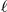 is the fragment length, *n*_f_ the average number of forks observed per fragments, and *t*_rep_ the replication time of the fragment. The replication time is given by 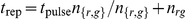, where 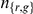 is the number of fully red (or green) labeled fragments while *n*_rg_ is the number of fully labeled fragments that have incorporated both labels (Demczuk et al., unpublished).) Thus, our fitted values nicely agree with the experiments. Second, the position of the second initiation zone, [1.11 Mb, 1.17 Mb] (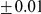 Mb), is almost completely located within the genomic deletion region of fragment 3, which is found between [1.12 Mb, 1.18 Mb]. (Remember that we did not use the deletion location to restrict the second initiation zone position while fitting.)

Our fit result has a reduced chi-square statistic of 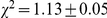 with 694 degrees of freedom. This high 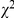 value is due to the simplistic initiation function we used. For example, a more complicated initiation function could be used to obtain a better fit of the red-green content profiles (e.g., we could use a higher initiation rate at the right side of fragment 2 or a different shape for the zone in fragment 3). Nevertheless, we believe that the simple replication scenario used here captures the most important features of the data set. Moreover, when we use the fit result to perform simulations of the SMARD experiment, we obtain statistics about the initiation/coalescence events and the replication time of each fragments that agree with the experimental values (Demczuk *et al.*, unpublished).

## Discussion

Over the years, various experimental approaches have been used to measure the absolute and relative efficiencies of origin firing in eukaryotic cells. However, the efficiency of origin firing does not encapsulate all the information required to understand how DNA origins of replication are regulated. Since eukaryotic genomes contain large numbers of origins, understanding their regulation requires a quantitative analysis of the dynamics of origin firing along the genome and across S phase. Achieving this goal requires comprehensive data sets about DNA replication across large genomic regions, as well as mathematical procedures for the analysis of complex data sets.

In this manuscript, we present a new set of rate equations that can be used to calculate the firing rate of DNA origin of replication using multiple sets of data (temporal order of replication, fork density, replication time). Our mathematical procedure is versatile and allows the analysis of complex data sets obtained using various experimental approaches (SMARD, microarrays, etc.). This is possible because our model follows the spatial and temporal evolution of several replication factors. In contrast, previous procedures have mostly relied on the analysis of individual parameters of DNA replication that can be modeled with limited detail (e.g., timing of replication). The main advantage of this technique is that the rate-equation solution corresponds to the exact mean-field replication program. Our approach thus provides more precise information about average replication kinetics than Monte Carlo simulations. It is faster, too. As discussed previously, simulation remains the appropriate technique for estimating statistical fluctuations of replication-related quantities. Since average replication kinetics is often the only information obtainable from experiments, our model is, in many practical cases, sufficient to reproduce experimental data. For these reasons, our mathematical procedure makes it possible to perform a faster, and more thorough, analysis of the process of DNA replication initiation and of its regulation in complex eukaryotes.

Although our procedure can be used to analyze data sets obtained with different experimental approaches, we validated it using results of recent SMARD experiments performed across a 1.4 Mb region which spans the mouse immunoglobulin heavy chain locus (Demczuk *et al.*, unpublished). We chose these experiments because, besides providing the data sets used in all the calculations, SMARD provided us with additional information that could be directly compared with the predictions of the procedure (e.g., the location of initiation events and fork collisions, the number of molecules containing such events, and the average number of events per molecules). The close match between calculated and experimental data sets indicates that our procedure can be used to make valuable inferences about various aspects of DNA replication in eukaryotes, with the calculations taking only modest computer resources. The usefulness of our model was illustrated by the series of fits of SMARD data we performed in Demczuk *et al.*, unpublished.

In Demczuk *et al.*, unpublished, the methods presented here implied that origin firing within the mouse Igh locus is compatible with the stochastic firing of origins throughout S phase, with a rate that varies along the locus. The Igh locus is divided into domains of similar firing rates, and the rate of firing within these domains is developmentally regulated. These observations contrast notably with results obtained in budding yeast, where the rate of firing varies from origin to origin and coordination in origin activity has not been observed [18]. Moreover, this approach allowed us to study various aspects of the developmental regulation of origin activity during B cell development.

In summary, the mathematical procedure described in this study has already provided new insights on the regulation of DNA replication initiation in mammalian cells and makes possible the study of additional phenomena such as replication time in the presence of fork velocities that depend on genome location or the impact of a correlation between initiation rates and fork density. Our method is thus a natural starting point for investigating checkpoint mechanisms where, for example, the cell regulates the local or global replication activity in response to various intra- or extracellular feedback signals.

## Supporting Information

Figure S1
**Space-time diagram of replication with inhomogeneous fork speeds.** The space-time point 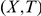 is replicated by an initiation that occurred within the shaded area (e.g., initiation A). By contrast, initiation B will replicate the location 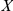 but only at a time 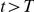. The inset defines symbols that refer to different portions of the shaded area. Note that 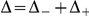.(TIF)

Supporting Information S1 Ending probability (homogeneous case). Modeling fork injection at boundaries.
(PDF)
