# Supplementary figures and images for: Modeling Inhomogeneous DNA Replication Kinetics
Source: PLoS One. 2012 Mar 7;7(3):e32053. doi: 10.1371/journal.pone.0032053 (PMC3296702; doi:10.1371/journal.pone.0032053)

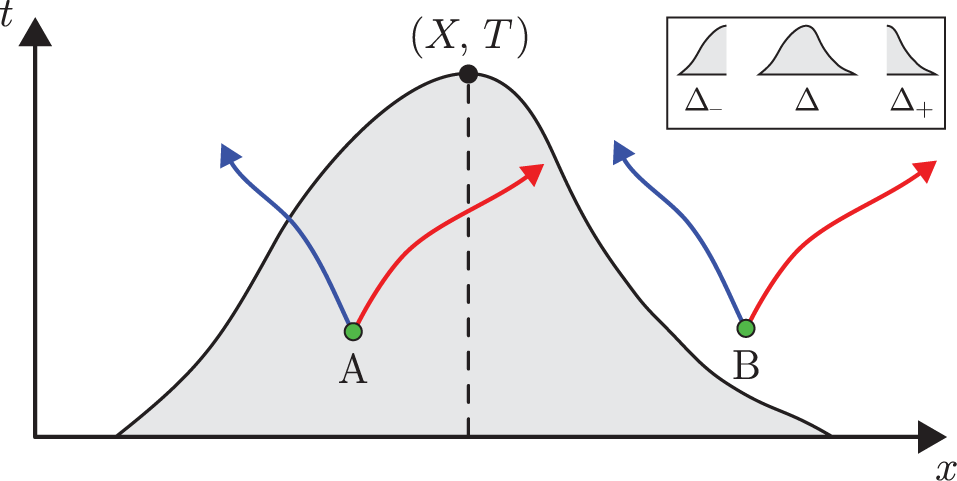

Supplement: Figure S1 — Space-time diagram of replication with inhomogeneous fork speeds. The space-time point is replicated by an initiation that occurred within the shaded area (e.g., initiation A). By contrast, initiation B will replicate the location but only at a time . The inset defines symbols that refer to different portions of the shaded area. Note that . (TIF) [file pone.0032053.s001.tif]
