# Supplementary material for: Modeling Inhomogeneous DNA Replication Kinetics
Source: PLoS One. 2012 Mar 7;7(3):e32053. doi: 10.1371/journal.pone.0032053 (PMC3296702; doi:10.1371/journal.pone.0032053)
Supplement: Supporting Information S1 — Ending probability (homogeneous case). Modeling fork injection at boundaries. (PDF) [file pone.0032053.s002.pdf]

# Supporting Information for: Modeling inhomogeneous DNA replication kinetics

Michel G. Gauthier [1], Paolo Norio[2,3], John Bechhoefer [1,*]
**1 Department of Physics, Simon Fraser University, Burnaby, British Columbia, Canada, V5A 1S6**
**2 Department of Oncology, Montefiore Medical Center, Moses Division, Bronx, NY, USA**
**3 Department of Medicine, Albert Einstein College of Medicine, Bronx, NY, USA**
∗ **E-mail: johnb@sfu.ca**

# Ending probability (homogeneous case)

To test our approximation of the replication ending probability $\widetilde{P}_{\rm e}(t)$ presented in Eq. 12, we consider here a test case where $P_{\rm e}(t)$ can be solved exactly—the case of homogeneous $I(x,t) = I$ and $v(x,t) = v$. For this case, we previously demonstrated that the replication fraction is given by [1]

$$f(t) = 1 - e^{-Ivt^2}\,, \tag{S1}$$

and the fork densities by

$$\rho_\pm(t) = \rho(t) = \frac{1}{2v}\frac{\partial f(t)}{\partial t} = Ite^{-Ivt^2}\,, \tag{S2}$$

where the genome length $L$ is assumed to be very large compared to $\sqrt{v/I}$. From Eq. 5, the coalescence time probability density function is given by

$$\phi_{\rm c}(t) = \frac{2v\rho(t)^2}{N_{\rm c}(1-f(t))} = \frac{4}{\sqrt{\pi}}\,(Iv)^{3/2}\,t^2 e^{-Ivt^2}\,, \tag{S3}$$

where the number of coalescences per cell cycle is given by $N_{\rm c} = \int_0^\infty \int_0^L \frac{2v\rho^2}{(1-f)}\,dx\,dt = L\sqrt{I\pi/v}/2$. The cumulative density function of the coalescence times, $\Phi_{\rm c}(t)$, is then

$$\begin{aligned}\Phi_{\rm c}(t) &= \int_0^t \phi_{\rm c}(t')dt' = \operatorname{erf}(\sqrt{Iv}t) - 2t\sqrt{\frac{Iv}{\pi}}e^{-Ivt^2} \\ &= \operatorname{erf}(t^*) - \frac{2t^*}{\sqrt{\pi}}e^{-t^{*2}} = \Phi_{\rm c}(t^*)\,,\end{aligned} \tag{S4}$$

where $t^* = \sqrt{Iv}t$ is a dimensionless time parameter. The probability that replication has finished by time $t^*$ can then be calculated as the probability that $N_{\rm c}$ coalescences have occurred by time $t^*$,

$$P_{\rm e}(t^*) = [\Phi_{\rm c}(t^*)]^{N_{\rm c}}\,. \tag{S5}$$

If $N_{\rm c}$ is large, we can re-write the exact solution as

$$\begin{aligned}P_{\rm e}(t^*) &= \exp\{-N_{\rm c}[1-\Phi_{\rm c}(t^*)]\} \\ &= \exp\left\{\frac{-2N_{\rm c}t^*e^{-t^{*2}}}{\sqrt{\pi}}\left[1 + \frac{\sqrt{\pi}\operatorname{erfc}(t^*)}{2t^*e^{-t^{*2}}}\right]\right\}.\end{aligned} \tag{S6}$$

This exact solution may be compared to our approximation in Eq. 12,

$$\widetilde{P}_{\rm e}(t^*) = \exp\left\{\frac{-2N_{\rm c}t^*e^{-t^{*2}}}{\sqrt{\pi}(1-e^{-t^{*2}})}\right\}. \tag{S7}$$

Both curves are sigmoidal functions interpolating between 0 and 1, as one expects. A numerical comparison of Eqs. S6 and S7 (not shown) shows that the maximum difference between the two expressions decreases as $N_{\rm c}$ increases.

## Modeling fork injection at boundaries

Here, we calculate the density of forks at the boundary of the region under study. The forks originate from the region outside the given boundary, and we thus do not have direct information about their origins. Figure S1 presents a space-time diagram of the replication situation assuming inhomogeneous initiation rates and the fork velocities. In this situation, the probability that an arbitrary point $X$ along the genome remains unreplicated at time $T$ equals the probability that no initiation occurred in the shaded space-time region shown in Fig. S1. That region is the set of space-time locations at which an initiation would passively replicate the point $(X, T)$ in the diagram. Thus, the replication fraction is given by

$$f(X,T) = 1 - \exp\left\{ - \iint_{x,t \in \triangle} I(x,t)\, dx\, dt \right\} , \tag{S8}$$

where $\triangle$ represents the shaded area in Fig. S1 (the shaded region has curved boundaries because of the inhomogeneity of the velocity profile). Using the point $X$ to define a boundary (dashed line) between two regions $\Delta_-$ and $\Delta_+$, the rate of replication at the boundary is the sum of two contributions

$$\begin{aligned} \frac{\partial f(X,T)}{\partial t} &= [1 - f(X,T)] \int_{x \in \triangle} I(x,t)\, dx\,, \\ &= [1 - f(X,T)] \int_{x \in \triangle_-} I(x,t)\, dx \\ &\quad + [1 - f(X,T)] \int_{x \in \triangle_+} I(x,t)\, dx\,, \end{aligned} \tag{S9}$$

where $\triangle_\pm$ represents the right $(+)$ and left $(-)$ portions of the shaded area in Fig. S1 (see legend). By analogy with Eq. 1, the fork densities at $X$ and $T$ are given by

$$\rho_\pm(X,T) = \frac{[1 - f(X,T)]}{v_\pm(X,T)} \int_{x \in \triangle_\mp} I(x,t)\, dx\,, \tag{S10}$$

where $\rho_\pm$ and $\triangle_\mp$ have opposite indices because right-moving forks at $x = X$ come from the left side of the shaded area and vice versa. Equations 14 and 15 are a special case (constant $I$ and $v$) of Eq. S10.

## References

1. Jun S, Zhang H, Bechhoefer J (2005) Nucleation and growth in one dimension. I. The generalized Kolmogorov-Johnson-Mehl-Avrami model. Phys Rev E 71: 011908.

**Supplementary Figure S1. Space-time diagram of replication with inhomogeneous fork speeds.** The space-time point $(X, T)$ is replicated by an initiation that occurred within the shaded area (e.g., initiation A). By contrast, initiation B will replicate the location $X$ but only at a time $t > T$. The inset defines symbols that refer to different portions of the shaded area. Note that $\Delta = \Delta_- + \Delta_+$.
